# The Associations Between Parental Burnout and Mental Health Symptoms Among Chinese Parents With Young Children During the COVID-19 Pandemic

**DOI:** 10.3389/fpsyt.2022.819199

**Published:** 2022-03-22

**Authors:** Minglong Chen, Yashuang Bai, Mingqi Fu, Ning Huang, Farooq Ahmed, Muhammad Shahid, Xiaohua Wang, Chengbin Liu, Xing Lin Feng, Jing Guo

**Affiliations:** ^1^Department of Health Policy and Management, School of Public Health, Peking University, Beijing, China; ^2^Center for Social Security Studies, Wuhan University, Wuhan, China; ^3^Department of Anthropology, Quaid-I-Azam University Islamabad, Islamabad, Pakistan; ^4^School of Insurance and Economics, University of International Business and Economics, Beijing, China; ^5^School of Social Development and Public Policy, Beijing Normal University, Beijing, China; ^6^School of Sociology, Huazhong University of Science and Technology, Wuhan, China

**Keywords:** parental burnout, mental health symptoms, family structure, family function, Chinese parents, COVID-19

## Abstract

The Coronavirus Disease 2019 (COVID-19) pandemic has caused numerous unexpected changes for families and societies, which have likely contributed to higher amounts of stress for most parents. This study aimed to examine the relationship between burnout and mental health among parents during the COVID-19. Pandemic exposure and household factors (e.g., family structure, family function) were examined as moderators. An online cross-sectional survey recruiting 1,209 adults was conducted from April 21st to April 28th, 2020 during the COVID-19 lockdown in China. The multivariable linear regression analysis was employed to test the association between burnout, household factors, and mental health among parents. Findings suggested that for parents with a young child, poorer mental health was related to a higher level of burnout (β = 0.220, *P* < 0.001) and greater exposure to the pandemic. Mothers of a single and/or young child had considerably poorer mental health. Moreover, the relationship between mental health and burnout among parents was significantly moderated by epidemic exposure (β = 2.561, *P* < 0.001), family structure (number of children: β = −1.257, *P* < 0.001; first child age: β=-1.116, *P* < 0.001) and family function (β = −0.574, *P* < 0.05). This study indicated that burnout symptoms were significantly associated with worse mental health among parents in China. Besides, exposure to the pandemic, family structure, and family function was found to moderate the association between burnout and mental health among parents. Therefore, the present study stressed enhanced access to mental health resources and emotional supports for parents during a public crisis to reduce the deleterious effects of burnout.

## Introduction

The coronavirus disease 2019 (COVID-19) outbreak spread rapidly throughout the country and quickly attracted global attention (1). To contain the infection spread, the Chinese government has issued nationwide emergency policies, with strict quarantine measures, including shutting down schools and non-essential businesses, and home quarantine. Those strict containment measures, severe economic loss, and great concerns regarding the virus infection all disrupted families' daily routines and stimulated overwhelmed pressures among families and society. A nationwide survey of psychological distress among Chinese people in the COVID-19 epidemic found that almost 35% of the respondents experienced psychological distress (2). In particular, during a prevalent pandemic lockdown, parents may experience extra pressures from family unemployment, income deduction, or the inability to work from home (3), as well as from home-schooling and parental communications (4).

Pressures from work and family are associated with an increased risk of parental burnout and push parents to be more vulnerable to mental health disorders during the pandemic (5). It should be noted that parental burnout differs from daily parenting stress because it is a prolonged response to chronic and overwhelming parental stress, with high risks and limited resources, and possibly followed by parental neglect and violent behaviors (5, 6). Existing studies pointed out that chronic stresses would deplete individuals' resources and lead to burnout symptoms if they last too long (7). Though gained little attention until recent years (8, 9), burnout was found to have significant impacts on mental wellbeing among parents (10, 11). An existing study has revealed that burnout was associated with higher levels of depressive symptoms, sleep disorders, as well as addictive behaviors among parents (12). In particular, unemployment, low levels of social support, and financial insecurity during the COVID-19 pandemic were found to place parents at a greater risk of burnout (13). For example, results from a survey conducted in Italy showed that the prevalence of parenting-related exhaustion (the main symptom of parental burnout) during the COVID-19 lockdown was as high as 17%, and greater parenting-related exhaustion was predicted by lower parental resilience, motherhood, having a child with special needs, and having younger children (14). Quasi-longitudinal research also revealed a higher parental burnout level during the pandemic lockdown than before including emotional distancing, exhaustion, and contrast (15). However, little was known about the relationship between burnout and mental health among Chinese parents during the COVID-19 pandemic. Moreover, these associations might vary between genders. According to the gender role theory (16), mother's mental health is more vulnerable to parenting issues than that of fathers, as they took more responsibilities in taking care of children. Simultaneously, mothers spent more time on primary childcare than fathers, and this gender inequality in the distribution of parental responsibilities and associated strains were linked to greater distress among mothers than fathers (17, 18). Indeed, mothers are more likely to regard the caregiving role as part of their social identity than fathers do and tend to ignore their own needs to meet society's expectations, therefore, are at an increased risk of becoming overwhelmed (19). As showed in recent research on the psychological wellbeing of parents, mothers had higher parental burnout and lower psychological wellbeing than the fathers during the prevalence of COVID-19 in Iran (20). Thus, burnout among mothers is likely to associate with higher mental health risks as compared to male caregivers.

Besides exploring the relationship between burnout and mental health among parents, this study further hypothesizes that several factors may work as moderators in this relationship. This first goes to the COVID-19 exposure. As noted by a cumulative risk model (21), the impact of burnout on mental health may be larger while the individuals are exposed to greater threats like the perceived impact of the pandemic. Meanwhile, the second one goes to family structure and functional factors (22). Regarding family structure, parents with more than two kids undoubtedly have to pay more time and energy to meet the extra parenting demands. A recent study in China indicated that mothers from two-kids families had higher parenting stress than their one-child counterparts (23), while Krieg (24) found that mothers in both one-child and two-child families reported equivalent levels of stress. Besides, age interval between siblings also accounts. One previous study proposed that mothers experienced greater stress during their kids' early childhood and their parenting stress would decrease as the kids became older (25). In addition, recent research found an increasing level of emotional symptoms such as frustration and sadness among mothers with pre-school children (from 2 to 5 years) during the pandemic (26). Another study conducted among Italian parents showed that parents of younger children experienced a higher level of parental stress as these children require continuative supervision and greater parental involvement (27). Thus, extra pressures in parenting more and younger kids may underdress parent's vulnerability in coping with burnout symptoms, and put them at higher risks of mental health disorders. Thirdly, the family functional factor might be a third moderator in the relationship between parental burnout and mental health. Impaired family functioning could contribute to decreased resources for the parental job (11), making parents more vulnerable to the consequence of burnout which occurs when resources are limited (6), thus leading to deteriorating mental health.

To date, emerging studies have investigated the effects of the COVID-19 crisis on parenting stress and the mental health of parents in China (28, 29). However, to our knowledge, no investigation has explored parental burnout, which differs from daily parental stress, and its relationship with psychological wellbeing among Chinese parents under this special background. In the present study, we administered a web-based survey of Chinese parents promptly to examine the relationship between burnout and parent mental health during the COVID-19 outbreak. Furthermore, prior research on burnout among parents have mainly focused on the risk factors analysis (10). For example, parents are at increased risk of burnout when they have prior psychiatric disorders, have lower emotional capabilities (30), have part-time work or off-work (10, 30), and lack social support (11). One existing study explored the consequence of burnout and found higher levels of escape, suicidal ideation, and other negative psychopathologies among parents with substantial burnout (5). This study tries to extend the post-burnout studies into traumatic context, and give a new perspective to evaluate the mental health burden of the COVID-19 pandemic on families and society.

### Aims and Hypothesis

The main objective of this study is to examine the relationship between burnout and mental health among parents in China. Then, we aim to explore the differences of this association between different genders. Finally, we want to further test if this association is moderated by pandemic exposure, family structure, and functional factor. On basis of the above-mentioned literature, three hypotheses were proposed. The first hypothesis is that parents with a higher level of burnout might be at greater mental health symptoms than their lower-leveled counterparts. The second hypothesis suggests that burnout among mothers is likely to associate with higher mental health risks, compared to fathers. Lastly, we assume that parents with higher traumatic exposure, having more and younger kids, and living with unhealthy family functions have higher levels of mental health disorders once they experienced levels of burnout.

## Methods

### Study Design and Participants

Data in this study were drawn from an online survey in April 2020, in China. During this time frame, governmental pandemic measures included: working remotely, keeping social distance, and closing schools and daycare centers. The questionnaires were distributed and retrieved through a web-based platform (https://www.wjx.cn/app/survey.aspx). A two-stage cluster sampling method was used to choose participants. In the first stage, three primary schools in Henan, Hubei, and Guangdong were selected. These schools were selected from the ordinary schools instead of special education schools, with the parents of children with special needs (e.g., developmental disabilities or physical illnesses) excluded. In the second stage, all students and their parents in selected schools contributed to a survey pool of this study. Headteachers helped to process the survey. Only parents with kid(s) aged 0 to 10 years were included in this study since they would experience a higher level of parenting stress due to the more parental assistance younger children often require. Participants were excluded if (1) they were unwilling to give informed consent; (2) The time to complete the questionnaire was <5 min; (3) We added quality control questions into the questionnaire. We excluded the questionnaires with obvious logical errors. According to a previous study, the incidence of various mental health problems among Chinese citizens during the epidemic was 20~35% (1). A sample size of 400 participants was required to achieve sufficient power to detect moderately sized associations (power = 0.80, r = 0.20, α = 0.05). The online survey required respondents to answer every question, so there was no missing data in our study. The final study sample consists of 1,286 participants. Participants received a small gift (e.g., 1–3 RMB) as a token of appreciation at the end of the session.

All participants joined the study voluntarily and gave written consent after being informed about the aim of the survey. This study was approved by the Ethics Committee of Peking University Medical Center and conducted in accordance with the ethical standards laid down in the 1964 Declaration of Helsinki and its later amendments.

## Measures

### Dependent Variables

Mental health was accessed by the Brief Symptom Inventory 18 (BSI-18, omitting suicidality) measuring somatization (6 items), depression (5 items), and anxiety (6 items), and a subset of 10 questions of the posttraumatic stress disorder (PTSD) checklist for DSM-5. All the questions were rated as “1 = never,” “2 = occasionally,” “3 = sometimes,” “4 = often,” “5 = Very often.” Since the four dimensions of mental health symptoms were highly correlated (ranging from 0.776 to 0.961), the total score of this scale was computed by averaging all 27 item scores. The higher the score, the poorer the mental health was. Confirmatory factor analysis supported this decision by indicating that one general psychopathology factor explained the correlational structure of the four latent psychopathology factors (RMSEA = 0.06; CFI = 0.974; SRMR = 0.043). The Cronbach's alpha of the scale in this study was 0.96.

### Independent Variables

Burnout among parents is assessed by the Parental Burnout Assessment (31). This scale includes 23 items in four dimensions (exhaustion, contrast with previous parental self, feelings of being fed up, and emotional distancing). All the items referred to general parenting. Specifically, in the case of multiple children, the questions referred to all their offspring (e.g., “I feel completely run down by my role as a parent,” “I don't think I'm the good father/mother that I used to be,” and “I can't stand my role as father/mother anymore”). Response options for each question are based on a 7-point Likert scale ranging from “never” to “every day.” Items were summed for a total score, with higher scores indicating a higher level of parental burnout. In this study, internal consistency for the total scale was 0.89, and for the four subscales were 0.91, 0.88, 0.84, and 0.63.

Exposure to COVID-19 was assessed with a question to describe if the subjects, family members, neighbors, or friend's exposure to COVID-19 pandemic, with “0” refers to “no,” while “1” denotes “yes.” Then, a total score was obtained by summing the scores of these items.

The family function was measured by the General Functioning 12-items (GF12) of The McMaster Family Assessment Device (FAD) (32), which has been validated as a single index measure to assess family functioning. The GF12 subscale is made up of 12 items, six items that reflect healthy family functioning and the other six items reflecting unhealthy functioning (33). Respondents could mark the level to which they agree with the statements with 1 to 4 points: 1 for completely disagree; 2 for disagree; 3 for agree; and 4 for completely agree. We calculated the score with inverse unhealthy item scores and the total score was the sum of these 12 items, with higher scores indicating fewer problems in a family's functioning. The internal consistency for this scale was 0.84.

### Socio-Demographic and the Family Structure

Based on previous related studies (34, 35), this study took the following demographic and socioeconomic characteristics into consideration: gender (male/female), age, province (Hubei/Henan/Guangdong/Else), occupation (manager/professional staff/individual/else), education level (high school and below/ college/undergraduate/master and above), marital status (married/others), family annual income (<100,000¥/100,000~200,000>¥/>200,000¥), first child age, number of children (one/two/more than two).

### Statistical Analysis

Data in this study were analyzed with the SPSS version 24.0. Descriptive statistics were calculated to describe the parental burnout, mental health of parents, exposure to COVID-19, family function, family structure (including the number of children and first child age), and other covariates. Means and standard deviations were used for continuous variables, and frequencies and percentages were computed for categorical variables. Main analyses included several multivariable linear regressions on mental health were conducted in three steps, with the same covariates used in each step: gender, age, province, occupation, family income level, and parental education level. In the first step, we examined the specific associations between parental burnout and mental health. Model 0 included every predictor separately to estimate its “raw” contribution to the mental health of parents. Model 1 put all the predictors into the model to determine the relationship between parental burnout and mental health. In the second step, the whole sample was divided into 2 groups by gender to examine gender differences in the effects of parental burnout on mental health. In the final step, interactions between parental burnout and the other three predictors (exposure to COVID-19, family structure, and family function) were examined in each model. Specifically, in Model 2 the interaction between parental burnout and COVID-19 exposure was included to examine its effect on parent mental health. Whereas, Model 3 included the interaction between parental burnout and number of children, Model 4 included the interaction between parental burnout and first child age, and Model 5 included the interaction between parental burnout and family function.

## Results

The socio-demographic characteristics of the study are presented in Table 1. Among the 1,286 participants, 74.2% of the surveyed parents were female. Nearly 22.4% were from Hubei, 14.6% from Henan, 42.0% from Guangzhou, and the remaining were from other provinces. In terms of the number of children, 46.2% of the parents had one child, 47.6% had two children, and 6.2% had three or more. Regarding the exposure to COVID-19, 18.2% reported that someone in their family, neighborhood, and friends had suffered from COVID-19. The average parental burnout score was 48.03 (SD = 21.60), and the mean overall family function score was 22.56 (SD = 5.42). The mean parental mental health score was 33.97 (SD = 11.44). More details are listed in Table 1.

**Table 1:** Parental burnout, family exposure, socio-demographic characteristics, and its binary relationship with mental health score among Chinese parents (*N* = 1,286).

**Variable**	**Frequency (***N***)**	**Percent (%)**	**B**	**Std.Err**	**β**
**Parent gender**					
Male	332	25.8			
Female	954	74.2	0.544	0.729	0.021
**Province**					
Hubei	288	22.4	−0.583	0.765	−0.021
Henan	188	14.6	0.986	0.903	0.030
Guangdong	540	42.0	−2.694	0.642	−0.116***
Else	270	21.0			
**Types of professionals**					
Manager	207	16.1	0.880	0.868	0.028
Professionals & technical	466	36.2	1.614	0.662	0.068*
Individual	194	15.1	−2.692	0.888	−0.084**
Else	419	32.6			
**Education level**					
High school and below	371	28.8	−3.385	0.698	−0.134***
college	237	18.4	−0.838	0.823	−0.028
undergraduate	460	35.8	1.206	0.665	0.051
Master and above	218	17.0			
**Marital status**					
Living with a partner	1,217	94.6	−2.731	1.414	−0.054
Others	69	5.4			
**Annual income**					
<100,000¥	790	61.4	−0.947	0.655	−0.040
100,000~200,000¥	266	20.7	1.203	0.787	0.043
>200,000¥	230	17.9			
**COVID-19 exposure**					
None	1,052	81.8			
Yes	234	18.2	7.005	0.804	0.236***
**Number of children**					
One	594	46.2	3.325	0.633	0.145***
Two	612	47.6	−2.611	0.635	−0.114***
More than two	80	6.2			
	**Mean**	**SD**			
Parental burnout scores	48.03	21.60	0.230	0.013	0.434***
Parent age	35.99	5.504	−0.158	0.062	−0.071*
First child age	6.791	2.368	−0.535	0.137	−0.110***
Family function (10–47)	22.599	1.041	−0.456	0.057	−0.216***
Mental health scores (27~135)	33.97	11.44			

*B, coefficient; Std.Err, standard error; β, beta; SD, standard deviation*;

*** *p < 0.001*,

** *p < 0.01*,

* *p < 0.05*.

Table 2 shows the results of the multivariable linear regression analysis for the relationship between parental burnout and mental health. In model 0, parental burnout, exposure to COVID-19, and family function were all significantly associated with mental health. In model 1, the family function became marginal significant to parents' mental health. Parents with younger children (β = −0.075, *P* < 0.01) have more mental health symptoms than their counterparts. Compared to parents with more children, parents with one child (β = 0.138, *P* < 0.05) have more mental health problems. Details can be found in Table 2.

**Table 2:** Multivariable linear regression analysis for the relationship between parental burnout and mental health score among Chinese parents (*N* = 1,286).

**Variable**	**Model 0**			**Model 1**		
	**B**	**Std.Err**.	**β**	**B**	**Std.Err**.	**β**
Burnout	0.227	0.013	0.429***	0.220	0.015	0.414***
Exposure	6.143	0.830	0.207***	5.322	0.781	0.179***
**Number of children (ref: > 2)**						
One child	3.353	1.372	0.146*	3.202	1.246	0.138*
Two children	0.947	1.345	0.041	1.658	1.212	0.072
First child age	−0.303	0.146	−0.062*	−0.364	0.130	−0.075**
Family function	−0.484	0.057	−0.229***	−0.090	0.059	−0.043*

*B, coefficient; Std.Err, standard error; ß, beta*;

* * p < 0.05*,

** * p < 0.01*,

*** * p < 0.001; Control variables: Gender, age, province, occupation, marital status, family income level*.

Table 3 displays gender differences in the relationship between parental burnout and mental health. Parental burnout (Male: β = 0.329, *P* < 0.001; Female: β = 0.453, *P* < 0.001) and epidemic exposure (Male: β = 0.246, *P* < 0.001; Female: β = 0.152, *P* < 0.001) are significantly associated with mental health for both males and females.

**Table 3:** Multivariable linear regression analysis for the relationship between parental burnout and mental health score among Chinese parents by gender.

**Variable**	**Male**			**Female**		
	**B**	**Std.Err**.	**β**	**B**	**Std.Err**.	**β**
Parental Burnout	0.192	0.032	0.329***	0.233	0.017	0.453***
COVID1-9 Exposure	7.823	1.639	0.246***	4.427	0.890	0.152***
Number of children (ref:>2)						
One child	3.269	3.134	0.130	3.288	1.347	0.147*
Two children	1.817	3.115	0.072	1.473	1.307	0.066
First child age	−0.470	0.280	−0.090	−0.364	0.147	−0.077*
Family function	−0.210	0.126	−0.090	−0.033	0.067	−0.016*

*B, coefficient; Std.Err, standard error; ß, beta*;

* *p < 0.05*,

*** *p < 0.001; Control variables: Gender, age, province, job, marital status, annual income*.

Table 4 reveals the relationships between four interactions and mental health (model 2–5). Participants who reported greater exposure to the COVID-19 and higher parental burnout showed elevated levels of mental health symptoms, while those who experienced higher parental burnout and parenting younger showed less mental health symptoms. Parents with more children and high family function would decrease the likelihood of developing mental health symptoms among parents with burnout symptoms.

**Table 4:** Multiple linear regressions for interaction effects of family exposure, demographic factors, and family function predicting parents' mental health.

	**Model 2**			**Model 3**			**Model 4**			**Model 5**		
	**B**	**Std.Err**.	**β**	**B**	**Std.Err**.	**β**	**B**	**Std.Err**.	**β**	**B**	**Std.Err**.	**β**
Parental Burnout	0.210	0.014	0.395***	0.219	0.015	0.411***	0.222	0.015	0.418***	0.207	0.016	0.389***
COVID-19 Exposure	4.938	0.748	0.166***	5.409	0.771	0.181***	5.297	0.772	0.178***	5.274	0.777	0.177***
Number of children	−1.695	0.488	−0.089***	−1.489	0.504	−0.078***	−1.554	0.505	−0.081***	−1.551	0.508	−0.081***
First child age	−0.369	0.124	−0.076***	−0.348	0.128	−0.072***	−0.342	0.128	−0.070***	−0.373	0.129	−0.077***
Family function	−0.082	0.057	−0.039	−0.095	0.058	−0.045	−0.094	0.059	−0.044	−0.109	0.060	−0.051
Parental Burnout*COVID-19 exposure	2.561	0.253	0.240***									
Parental Burnout*number of children				−1.257	0.267	−0.114***						
Parental Burnout*first child age							−1.116	0.276	−0.099***			
Parental Burnout*family function										−0.574	0.293	−0.053*

*B, coefficient; Std.Err, standard error; ß, beta*;

* *p < 0.05*,

*** *p < 0.001; Control variables: Gender, age, province, job, marital status, annual income*.

## Discussion

This study examined the relationship between burnout and mental health among Chinese parents during the COVID-19 pandemic. In this relationship, we further tested the moderating role of exposure to the COVID-19, family structure, and family function. Our findings suggested that experiencing burnout, having greater exposure to the pandemic were related to worse mental health symptoms among parents. Mothers with one child or young children had worse mental health symptoms. Besides, the relationship between burnout and mental health among parents was significantly moderated by the level of epidemic exposure, the family structure, and the wellbeing of family function. Greater exposure to the pandemic enhanced the relationship between burnout and more mental health symptoms. On the contrary, parents with older-aged and/or more than one kid, and/or reported healthy family function are less likely to develop mental health symptoms despite burnout.

Firstly, burnout is significantly associated with mental health among parents, which is in line with previous studies (10, 36). These studies have indicated a high level of stress and low mental wellbeing among parents who experienced parental burnout caused by prolonged exhaustion from parenting tasks (6, 36). According to the transactional model of stress, a sense of burnout among parents might evolve into one specific chronic stress (37), while overburden pressure could lead to poor psychological adjustments and more mental health problems (38). Meanwhile, the Job Demand-Resources (JD-R) model (39) posits that job burnout occurs when job demands are high and job resources are limited. Alike, parental burnout develops when more parenting needs are not compensated by enough resources (40). Poor access to parental resources might cause frustration and disengagement among parents, and result in exhaustion and other mental health impairments potentially (6). Moreover, in Chinese society, families generally outsource care resorting to after-school training institutions and grandparents that can help with the education and caring of their children. However, during the lockdown, all these external supports were limited and parents had to run childcare tasks and newly acquired family issues themselves, which made them exhausted from parenting tasks and reduced their mental health (28). Taken together, burnout among parents could be either a factor of acute mental health disorders during the pandemic or a signal of long-term mental health problems after the trauma. We urge that more attention should be paid to burnout symptoms among parents in China.

Secondly, the correlations between parental burnout and mental health are significant in both father and mother groups. In agreement with previous studies (20, 41), we found that mothers were more vulnerable to mental distress than fathers owing to parenting issues, which reflects that female takes most of the home care responsibilities in China. In addition, these results are in line with other studies that highlight COVID-19 could bring additional gender burdens, with women experiencing increased vulnerability and low psychological wellbeing (42, 43). Thus, we propose that more services in mental health protection should be delivered to females in a household context.

Besides, this study reveals that having a younger and only one child was associated with an elevated level of mental health symptoms among mothers, yet healthier family function played an inverse connection. It is obvious that younger kids compared with their older counterparts need more intensive family care, and produce greater parenting stress among parents (25). However, it is counterintuitive that parents with only one child are with more mental health problems than those parenting more kids. Possibly, parents in China still hold a traditional belief that “more children indicate more happiness,” thus a greater number of kids in their family are helping to shape a sense of happiness and resilience (44). In Guangdong province, in particular, the number of kids often indicates the level of life satisfaction under the local “Zongci” culture. Parents could obtain more emotional support from their children in multi-children family (45). When it comes to a family function, an existing study found that an unhealthy family function may lead to marital conflicts and eventually to depression among family members (46). A healthy family function, on the contrary, would work with a sense of life satisfaction and hopefulness, and serve to protect mental health (47).

Thirdly, this study suggests that the relationship between burnout and mental health was significantly moderated by epidemic exposure, family structure, and family function. Previous studies also showed a high level of traumatic exposure forced parents into a more frightened and fragile condition, which lowers their threshold of burdening the burnout sensation (14, 15). On the other hand, burnout, as a sense of exhaustion or a result of long-term stress, makes parents more vulnerable to the following negative life events and exacerbates their capabilities to cope with the potential negative affections. Meanwhile, family structure with more and older children reduced the risk of mental health problems among parents with burnout. A possible explanation is that children being older-aged and with a sibling(s) are more probable to care for others, and more likely to provide social support inversely to the parents once burnout emotions existed (25, 48). Thus, this study proposes that reducing the level of traumatic exposure and/or giving voice to a healthy family function might be the interesting starting point in mental health protection among parents in China.

## Limitations and Strengths

Several limitations of this study should be acknowledged. First, apart from the covariates mentioned in this study, there are yet many other factors such as living arrangements that have not been controlled in this study. In addition, the measure of COVID-19 exposure is not detailed and important information (e.g., severity and duration of symptoms) are overlooked. Moreover, gender differences are addressed but parents in the study were not couples and dyadic processes (co-parenting, coping, division of labor, the degree of caregiving involvement) are not addressed. Second, the parents of children with special needs (e.g., developmental disabilities or serious physical illnesses) were excluded in our study, whose mental health might be worse due to a higher level of parental burden. The samples including parents of children with developmental disabilities or physical illnesses are expected in future research to examine the child-related predictors on parents' mental health. Third, the sampling methods used in our study were not based on a random selection, which might constrain the generalizability of our findings. Finally, with the cross-sectional design of the current research, it is hard to ensure the direction of causal relationships among the major variables tested in the model, though the theoretical framework has provided full support for these hypotheses. Longitudinal data are expected in future research to help clarify the relationship patterns.

Despite these limitations, there are several implications for practitioners that can support the parents during the COVID-19 difficulties. First of all, urgent consideration should be given to how additional support can be provided to Chinese parents experiencing burnout during the COVID-19 pandemic context. Applicable and proactive interventions, and family education programs, for example, can be proposed. Meanwhile, given the direct and moderating effect of family function in mental health inflammation, community service to help to facilitate better family communications and increase life satisfaction should be encouraged. Last but not the least, it is vital to identify what advice and support could help parents most according to their different situations during the COVID-19 lockdown. In detail, for those women who have only one child or parenting younger children, more effective strategies to prevent burnout and more support of childcare may effectively reduce mother's parenting stress and therefore be beneficial to their mental health.

## Conclusion

This study indicates that burnout symptoms are significantly associated with worse mental health among parents in China. It also finds out the relationships between parental burnout and mental health differs across gender. Females are more vulnerable to parenting-related pressures than their male counterparts. Besides, exposure to the pandemic, family structure, and family function is found to moderate the association between burnout and mental health among parents. This study urges that community services and target interventions with a healthy family structure and function might be beneficial to improve parent's mental health.

## Data Availability Statement

The original contributions presented in the study are included in the article/supplementary material, further inquiries can be directed to the corresponding authors.

## Ethics Statement

The studies involving human participants were reviewed and approved by Peking University. The patients/participants provided their electronic informed consent to participate in this study.

## Author Contributions

JG designed the study. MC and YB drafted the manuscript and analyzed the data. MF, NH, FA, MS, XW, CL, and XLF were involved in revising the manuscript. All authors were involved in writing the manuscript and approve of its final version.

## Funding

This work was supported by the National Social Science Fund of China (Grant Number: 20VYJ042) to JG.

## Conflict of Interest

The authors declare that the research was conducted in the absence of any commercial or financial relationships that could be construed as a potential conflict of interest.

## Publisher's Note

All claims expressed in this article are solely those of the authors and do not necessarily represent those of their affiliated organizations, or those of the publisher, the editors and the reviewers. Any product that may be evaluated in this article, or claim that may be made by its manufacturer, is not guaranteed or endorsed by the publisher.
